# Effects of acute systemic inflammation on the interplay between sad mood and affective cognition

**DOI:** 10.1038/s41398-017-0043-0

**Published:** 2017-12-11

**Authors:** Sven Benson, Alexandra Brinkhoff, Larissa Lueg, Till Roderigo, Andreas Kribben, Benjamin Wilde, Oliver Witzke, Harald Engler, Manfred Schedlowski, Sigrid Elsenbruch

**Affiliations:** 1 Institute of Medical Psychology and Behavioral Immunobiology, University Hospital Essen, University of Duisburg-Essen, Essen, Germany; 2 Department of Nephrology, University Hospital Essen, University of Duisburg-Essen, Essen, Germany; 3 Department of Infectiology, University Hospital Essen, University of Duisburg-Essen, Essen, Germany

## Abstract

Experimental endotoxemia is a translational model to study inflammatory mechanisms involved in the pathophysiology of mood disorders including depression. Disturbed affective cognition constitutes a core aspect in depression, but has never been studied in the context of inflammation. We combined experimental endotoxemia with an established experimental mood induction procedure to assess the interaction between acute inflammation and sad mood and their effects on affective cognition. In this randomized cross-over study, *N* = 15 healthy males received endotoxin (0.8 ng/kg lipopolysaccharide iv) on one study day and placebo an otherwise identical study day. The affective Go/Nogo task was conducted after experimental induction of neutral and sad mood. Inflammatory markers were assessed hourly. Endotoxin application induced a transient systemic inflammation, characterized by increased leukocyte counts, TNF-alpha and interleukin-6 plasma concentrations (all *p* < 0.01, interaction effects). Mood induction led to greater sadness ratings, with highest ratings when sad mood was induced during inflammation (*p* < 0.05, interaction effect). Based on a 2 (endotoxin vs. placebo) × 2 (sad vs. neutral mood) × 2 (sad vs. happy Go/Nogo target words) factorial design, we observed a significant target × endotoxin condition interaction (*p* < 0.01) reflecting slower responses to sad targets during endotoxemia. Additionally, we found a valence × mood interaction (*p* < 0.05), reflecting slower reaction times to sad targets in sad mood. In summary, acute inflammation and sad mood are risk factors for disturbed affective cognition. The results may reflect a mood-congruency effect, with prolonged and sustained processing of mood-congruent information during acute inflammation, which may contribute to depression risk.

## Introduction

Systemic inflammation is increasingly recognized as a risk factor for depression^1–3^. Major depression is highly prevalent in chronic inflammatory conditions, and a substantial proportion of patients with major depression exhibit elevated concentrations of circulating inflammatory mediators such as pro-inflammatory cytokines^4–6^. Further support comes from clinical trials, which documented improved mood in response to anti-inflammatory treatment^2,7^. As a translational model, experimental endotoxemia has made a significant contribution to elucidating the mechanisms underlying the effects of pro-inflammatory mediators on the behavioral and neural correlates of depression symptoms. In healthy individuals, a number of depression-like symptoms can reliably and dose dependently be induced by application of bacterial endotoxin such as lipopolysaccharide (LPS)^8^, including negative mood^9–14^, disturbed psychosocial functioning^15–18^ and altered reward processing^13^. In the human LPS model, we were recently able to show for the first time that the pro-inflammatory cytokine interleukin-6 (IL-6) was increased in cerebrospinal fluid (CSF). Increased IL-6 concentrations in CSF were significantly correlated with dysthymia, supporting the role of central cytokines in dysthymia with broad implications for the pathophysiology of depression in patients^9^.

Disturbed affective cognition constitutes a core aspect in depression^19^, but has never been studied in the context of inflammation. The concept of affective cognition refers to the cognitive processing and evaluation of emotionally salient information^19^. Disturbed affective cognition reflects “deficits at the interface between affect and cognition”^19^, resulting in negative biases in emotion categorization, or biases toward negative information or sad stimuli^19,20^. According to the cognitive theory of depression^21^, such attentional biases do not merely constitute an important symptom in depressed patients^22^, but have also been related to depression risk, exacerbation and maintenance of symptoms^23^. Clinically depressed patients, individuals at risk for depression and even healthy individuals in sad mood states^24,25^ demonstrably display biases in the cognitive processing of emotionally salient information^20,22^. Although negative biases for emotional and social stimuli have been shown in response to acute inflammation^26^, virtually nothing is known about affective cognition during inflammatory states. Experimental mood induction paradigms allow to investigate the interplay between mood and cognition^23^ and allow insights into the question how mood impacts on affective cognition in healthy volunteers^24^.

We present the first study that experimentally tests biases in affective cognition, assessed with the established affective Go/No-Go task^27^, during human endotoxemia. By combining LPS administration with a mood induction paradigm, we specifically hypothesized that sad mood and inflammation would facilitate the processing of mood-congruent stimuli in the affective Go/Nogo task (i.e., shorter response latencies for negative compared with positive target words, lower number of response errors), with greatest effects when sad mood is induced during endotoxemia.

## Materials and methods

### Recruitment and safety routine

Healthy male volunteers aged 18–40 years were recruited by public advertisement. The screening and safety procedures consisted of a physical examination, a personal interview conducted by a physician and laboratory assessments (i.e., complete blood cell count, liver enzymes, renal parameters, electrolytes, coagulation factors and C-reactive protein), which were conducted before, 24 h after endotoxin administration and up to 1 week after completion of the study. General exclusion criteria were any pre-existing or current physical or psychiatric illness including sleep disturbances like insomnia, body mass index (BMI) < 19 or ≥ 29 kg/m^2^, current medications, smoking, regular alcohol use ( > 4 drinks per week) and depression scores exceeding published cut-offs of the Beck Depression Inventory^28^. Participants received instructions to maintain regular night sleep before study days, and activities interfering with regular sleep such as night shifts were exclusionary. Further, participants were not allowed to drive a vehicle on study days. The study was conducted in accordance with the Declaration of Helsinki and was approved by the Institutional Ethics Review Board of the Medical Faculty of the University of Duisburg-Essen (approval no. 15-6533-BO). Signed informed consent was obtained and participants received financial compensation for their participation in the study.

### Study design

This randomized, double-blind, placebo-controlled, cross-over study was comprised of two identical study days for each participant, that is, an endotoxin and a placebo condition, which were conducted in a randomized and counterbalanced order (www.randomizer.org used for randomization). For study design, see Suppl. Fig. 1. Upon arrival at the laboratory, participants first underwent a medical check-up and were then prepared for the study. An intravenous catheter was inserted into a forearm vein of the non-dominant arm for repeated blood collection and LPS/placebo injection, respectively. After a 30-min resting period, a baseline blood sample was obtained. After 30 min, participants received an intravenous injection of either LPS (endotoxin condition) or physiological saline (placebo condition) (see below). Aiming to minimize the impact of circadian changes in neuroendocrine parameters such as cortisol, participants were injected between 12 p.m. and 2 p.m., at identical time points on both study days. The time interval between study days was at least 7 days. Two to 4 h post injection of LPS or placebo, a neutral and a sad mood condition were completed on each study day. Both mood conditions were comprised of the experimental induction of neutral or sad mood (see below), immediately followed by affective Go/Nogo tasks (AGNG; see below). Note that the order of mood conditions (neutral followed by sad) was intentionally not counterbalanced given the temporal dynamics of the LPS-induced inflammatory response, as previously accomplished^29,30^. A fixed order is advantageous given greater inter-individual comparability of plasma cytokine levels and in order to avoid possible carry-over effects from sad to neutral mood conditions.

Blood for cell counts and cytokine analyses was collected at a baseline, as well as 1, 2, 3, 4 and 6 h after injection. Following each blood draw, body temperature (with an intraaurical thermometer), blood pressure (Dinamap Compact T, Critikon, Norderstedt, Germany), heart rate (pulse oximetry; Kernmed Oled, Ettlingen, Germany) self-reported mood and physical sickness symptoms (with standardized questionnaires, see below) were assessed. Investigators who conducted the mood induction and AGNG were blinded to the study condition.

### Experimental induction of systemic inflammation

To induce an acute systemic inflammatory response, participants received an intravenous injection of 0.8 ng endotoxin per kilogram of body weight dissolved in sterile water (LPS condition). This dose has been demonstrated to reliably induce an increase in pro-inflammatory cytokines 1–4 h post-injection in previous studies of our group^10,11,31^. The LPS used herein (reference standard endotoxin from *Escherichia coli*, serotype O113:H10:K-negative, lot H0K354, United States Pharmacopeia, Rockville, MD, USA) had been subjected to a microbial safety testing routine approved by the German Federal Agency for Sera and Vaccines (Paul Ehrlich Institute, Langen, Germany). In the placebo condition, participants received an equivolume injection of physiological saline (sterile, pyrogen-free isostonic NaCl solution, B Braun Melsungen, Melsungen, Germany).

### Mood induction procedure

On both study days, sad and neutral emotional states were induced using a modified Velten mood induction procedure^32^ according to^33^. In both mood conditions, volunteers were exposed to a series of 30 Velten statements, which were presented on a computer screen for at least 24 s until participants could press the space bar to move on to the next sentence. Participants had been instructed to attempt experiencing the mood suggested by the statements. To intensify the mood induction effect, participants listened to mood-congruent music via noise-canceling headphones (Sennheiser, Wennebostel, Germany). Prior to the initiation of Velten statements, two different pieces of music (Samuel Barber: *Opus 11, Adagio for Strings*; Tomaso Albinoni: *Adagio in G Minor*) were presented for 1 min and rated. The piece, which was rated as more sadness inducing, was then presented during the sad mood condition. During the sad mood condition, participants were exposed to 30 self-referent negative statements (e.g., “I've felt so alone before that I could have cried”). In the neutral mood condition, participants were exposed to 30 neutral statements (e.g., “The Chinese language has many dialects, including Cantonese, Mandarin, and Wu”) while listening to Gustav Holst: *Opus 320 The Planets, VII. Neptune, the Mystic*. Both mood conditions lasted approximately 18 min, and sad mood was rated on visual analog scales (VAS, anchors “not at all” to “very much”) at the beginning and the end of each condition.

### Affective Go/Nogo task

The AGNG task^27^ was started immediately after induction of sad or neutral mood. Participants were seated in an upright position in a hospital bed, with a fixed distance to a notebook placed in front of them and were wearing noise-canceling headphones. Stimuli consisted of happy and sad German words extracted from a published database, which offered words rated and categorized by valence^34,35^. Happy and sad words were matched for word length (i.e., number of letters). The AGNG task comprised 10 blocks of 18 valenced words (9 happy and 9 sad), with rest phases between blocks. Before each block, participants were instructed via the computer screen to respond either to happy (H) or to sad (S) words by pressing the space bar as fast as possible. A 450 Hz tone (500 ms) sounded for each error, but not for omissions. The task comprised two practice blocks followed by eight test blocks, with targets for the 10 blocks presented in a HHSSHHSSHH or SSHHSSHHSS order. Thus, four test blocks were “shift” blocks due to the shift in target valence (shift from happy to sad or sad to happy target words), and four blocks were “non-shift” blocks because the target valence was unchanged. Each word was presented for 300 ms, with a 1500-ms interstimulus interval. Reaction times < 150 or >1500 ms were considered as outliers and excluded before analysis^27^. Mean reaction times to correct responses, numbers of commission (responses to distractors), and omission (non-response to targets) errors were calculated as performance measures according to^27^.

### Assessment of inflammatory markers, self-reported mood and sickness symptoms

Inflammatory parameters, mood and physical sickness symptoms were assessed before (baseline) as well as 1, 2, 3, 4 and 6 h after endotoxin or placebo injection. Blood for cytokine analyses and blood cell counts was collected in EDTA-treated tubes (S-Monovette, Sarstedt, Nümbrecht, Germany). Plasma was immediately separated by centrifugation (2000 *g*, 10 min, 4 °C) and stored at −80 °C until analysis. Plasma Tumor Necrosis Factor (TNF)-α and IL-6 concentrations were measured by enzyme-linked immunosorbent assay (ELISA) (Human Quantikine ELISA, R&D Systems, Minneapolis, MN, USA) according to the manufacturer’s protocols. Assay sensitivity was 0.70 pg/ml. Complete blood counts including white blood cell (WBC) differential were obtained using an automated hematology analyzer (XP-300, Sysmex Europe, Norderstedt, Germany). Positive mood (euthymia) and negative mood (dysthymia) were assessed with two five-item subscales of the state version of the standardized and validated German State Trait Anxiety Depression Inventory (STADI) as previously accomplished^9^. Physical sickness symptoms were measured using an adaption of the validated General-Assessment-of-Side-Effects (GASE) questionnaire^36^ as previously described^10^. Briefly, subjects rated the present severity of 17 different physical symptoms from 0 (“not present”) to 3 (“severe”), and sum scores were calculated.

### Statistical analyses

Data analysis was performed using SPSS 22.0 (SPSS Inc., Chicago, IL, USA) and the level of significance was set at α < 0.05. Normal distribution of variables was tested using Kolmogorov–Smirnov test, and non-normally distributed variables (i.e., cytokines) were log-transformed prior to analysis. Data are shown as mean ± standard error of the mean (SEM). Sample size was based on previous experiments implementing a cross-over design, and provided sufficient statistical power to detect at least large effects in analysis of variance (ANOVA; 1-β = 0.82 for ANOVA interaction effects as calculated with G-Power, version 3.1.9.2). Endotoxin effects on physiological measures, mood and physical sickness symptoms were analyzed by repeated-measures analysis of variance (rm-ANOVA) with endotoxin condition (LPS vs. placebo) and time as within-subject factors, followed by Bonferroni-corrected post-hoc paired *t-*tests (two-tailed) in case of significant interaction effects. To analyze performance measures in the AGNG task (i.e., reaction times, number of commission and omission errors), rm-ANOVA was calculated as a “full model” with endotoxin condition (LPS vs. placebo), mood condition (sad vs. neutral mood), shift condition (shift vs. non-shift blocks) and valence (sad vs. happy target words). In addition, we computed separate rm-ANOVAs within shift and non-shift conditions with the factors endotoxin condition, mood condition, and valence. Finally, we tested the interaction of endotoxin and sad mood conditions using delta reaction times for positive vs. negative target words (with positive scores indicating a tendency to respond slower and negative scores indicating a tendency to respond faster to sad targets) according to Murphy et al.^27^. Delta reaction times were compared with rm-ANOVA with the factors endotoxin and mood, followed by post-hoc paired *t-*tests (two-tailed). To account for putative inter-correlations between mood and sickness symptoms, analyses of mood induction and affective Go/Nogo data were additionally conducted with physical sickness symptom scores as covariate (computed as delta of GASE scores between endotoxin and placebo condition at 3 h post injection).

## Results

### Sample characteristics

The study sample consisted of healthy male volunteers (*N* = 15) with a mean age of 26.2 ± 1.1 years and a mean BMI of 24.4 ± 0.6 kg/m^2^. The majority of participants (*N* = 13, 86.7%) had > 12 years of formal education (German Abitur). Beck depression inventory scores were low and within the normal range (2.6 ± 0.8).

### Effects of endotoxin on inflammatory parameters, mood and sickness symptoms

**Fig. 1:**
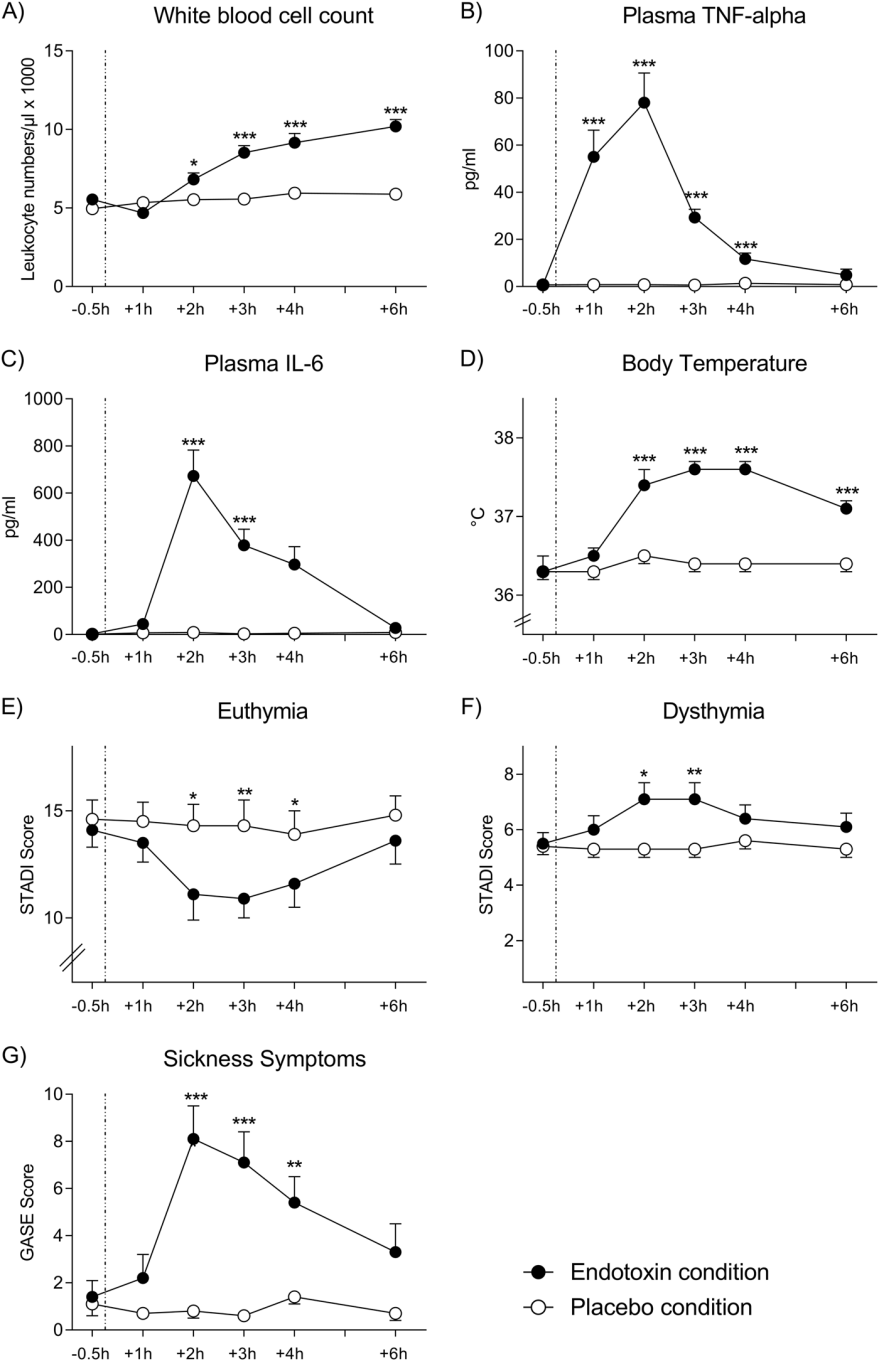
**Effects of endotoxin** WBC counts **a**, plasma TNF-alpha **b**, IL-6 **c**, body temperature **d**, as well as euthymia **e**, dysthymia **f**, and physical sickness symptoms **g** were repeatedly measured at baseline and up to 6 h post injection of endotoxin (LPS, 0.8 ng/kg body weight) or saline (placebo). LPS administration led to significant increases in WBC counts, plasma IL-6, salivary cortisol, body temperature, indicating a systemic immune activation, as well as to reduced euthymia and increases in dysthymia and the number and intensity of physical sickness symptoms. *p < 0.05, **p < 0.01, ***p < 0.001; results of Bonferroni-corrected post-hoc paired *t*-tests. For ANOVA results, see text. STADI State Trait Anxiety and Depression Inventory; GASE General-Assessment-of-Side-Effects questionnaire

Endotoxin administration induced an acute and transient systemic inflammatory response. Compared with the placebo condition, WBC counts (F_(5, 70)_ = 35.9, *p* < 0.001, ƞ_p_² = 0.72), as well as plasma TNF-α (F_(5, 70)_ = 71.7, *p* < 0.001, ƞ_p_² = 0.84) and IL-6 (F_(5, 70)_ = 65.3, *p* < 0.001, ƞ_p_² = 0.82) concentrations showed significant increases in response to endotoxin, along with a slight, but significant rise in body temperature (F_(5, 70)_ = 29.4, *p* < 0.001, ƞ_p_² = 0.69) (all ANOVA interaction effects of time × endotoxin condition, see Fig. 1 for results of post-hoc testing). Further, self-reported dysthymia significantly increased (STADI score; F_(5, 70)_ = 4.8, *p* < 0.05, ƞ_p_² = 0.28), whereas euthymia decreased (STADI score; F_(5, 70)_ = 2.3, *p* = 0.05, ƞ_p_² = 0.16) in response to LPS application (both ANOVA time × endotoxin condition interactions, see Fig. 1 for results of post-hoc testing). Finally, LPS application induced a significant increase in physical sickness symptoms, with highest scores 2–3 h post injection (F_(5, 70)_ = 10.7, *p* < 0.001, ƞ_p_² = 0.43, see Fig. 1 for results of post-hoc testing).

### Mood induction

**Fig. 2:**
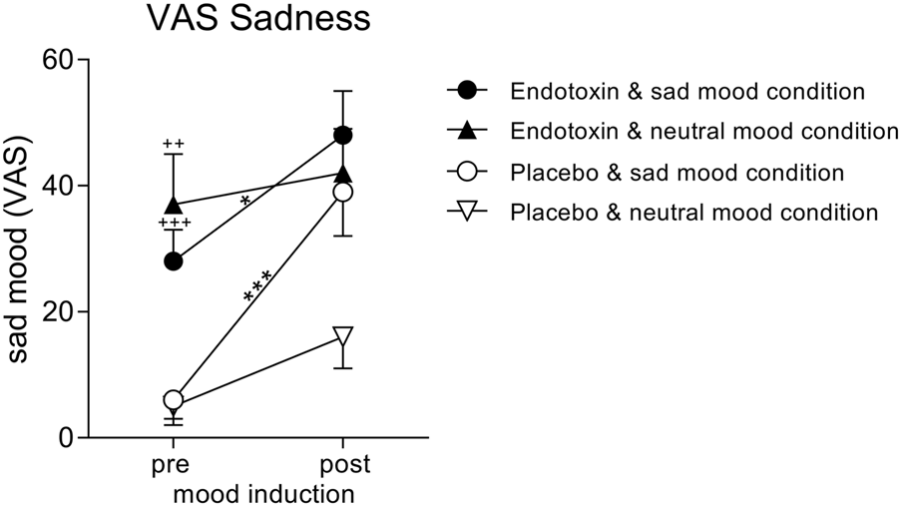
**Effects of mood induction procedure on VAS mood ratings** Sadness was measured with a VAS before (pre) and after (post) the experimental induction of sad or neutral mood during the endotoxin and the placebo condition. The induction of sad mood led to significant increases in sadness ratings both within the endotoxin and the placebo condition (*p < 0.05, ***p < 0.001, results of Bonferroni-corrected post-hoc paired* t*-tests). In addition, participants showed higher baseline sadness ratings (i.e., before the mood induction paradigm started) within the endotoxin condition (^++^ *p* < 0.01, ^+++^ *p* < 0.001, results of Bonferroni-corrected post-hoc paired* t*-tests), reflecting endotoxin effects on sadness ratings. For ANOVA results, please see text

The induction of sad mood led to the expected increase in VAS sadness ratings (F_(1, 13)_ = 10.3, *p* = 0.007, ƞ_p_² = 0.44, ANOVA main effect of mood condition). Sadness ratings were higher within the endotoxin condition (F_(1, 13)_ = 24.1, *p* < 0.001, ƞ_p_² = 0.65, ANOVA main effect of endotoxin condition), which reflects endotoxin effects on mood ratings. Finally, increases in sadness ratings were more pronounced within the placebo compared with endotoxin condition (F_(5, 70)_ = 6.0, *p* = 0.029, ƞ_p_² = 0.31, interaction of endotoxin × mood condition), which is attributable to higher baseline sadness ratings within the endotoxin condition (see Fig. 2 for results of post-hoc testing). To explore possible inter-correlations between mood and sickness symptoms, we repeated analysis with physical sickness symptom score as a covariate. In support of this notion, only the effect of endotoxin condition remained significant (F_(1, 13)_ = 5.9, *p* = 0.03, ƞ_p_² = 0.33).

### Affective Go/Nogo

For mean reaction time as main outcome variable in the AGNG (Table 1), rm-ANOVA revealed no significant endotoxin × mood × valence × shift interaction effects in the full model (F_(1, 14)_ = 0.1, *p* = 0.75, ƞ_p_² = 0.01). Importantly, mean reaction times did not differ significantly between endotoxin and placebo conditions (F_(1, 14)_ = 0.1, *p* = 0.81, ƞ_p_² = 0.01, main effect of endotoxin condition), indicating that endotoxin administration *per se* did not affect response time.

**Table 1:** Affective Go/NoGo test

		LPS condition		Placebo condition	
		Neutral mood	Sad mood	Neutral mood	Sad mood
Shift blocks					
Reaction time in ms	Happy targets/sad distractors	728.5 ± 92.2	724.1 ± 97.2	764.5 ± 91.8	733.4 ± 106.5
(mean ± SD)	Sad targets/happy distractors	751.4 ± 127.1	760.9 ± 127.4	744.1 ± 89.5	722.9 ± 113.7
Total commission (distractor) errors	Happy targets/sad distractors	1.47 ± 1.13	1.00 ± 1.07	1.07 ± 1.28	1.40 ± 1.40
(mean ± SD)	Sad targets/happy distractors	1.27 ± 1.16	0.8 ± 0.86	1.00 ± 0.93	1.33 ± 1.35
Total omission (target) errors	Happy targets/sad distractors	1.80 ± 1.52	1.40 ± 1.50	1.33 ± 1.18	1.80 ± 1.61
(mean ± SD)	Sad targets/happy distractors	2.73 ± 2.37	2.53 ± 1.13	2.27 ± 0.80	2.13 ± 1.00
Non-shift blocks					
Reaction time in ms	Happy targets/sad distractors	755.3 ± 120.8	714.8 ± 86.2	750.7 ± 111.6	707.9 ± 125.8
(mean ± SD)	Sad targets/happy distractors	713.9 ± 106.1	715.8 ± 105.0	726.1 ± 89.6	737.8 ± 109.9
Total commission (distractor) errors	Happy targets/sad distractors	0.80 ± 0.86	0.93 ± 1.03	1.20 ± 1.01	1.20 ± 1.08
(mean ± SD)	Sad targets/happy distractors	0.8 ± 1.08	0.93 ± 1.22	1.00 ± 1.00	1.07 ± 1.10
Total omission (target) errors	Happy targets/sad distractors	1.7 ± 1.10	2.20 ± 2.62	1.33 ± 0.62	1.47 ± 0.91
(mean ± SD)	Sad targets/happy distractors	1.20 ± 0.96	1.47 ± 1.41	1.27 ± 0.88	1.67 ± 1.23

Mean and SD for reaction time in milliseconds (ms), and total numbers of commission (distractor) and omission (target) errors, separated for shift and non-shift blocks. For results of repeated-measures analyses of variance, see text

Given that shift condition significantly interacted with endotoxin and valence conditions (F_(1, 14)_ = 15.0, *p* = 0.002, ƞ_p_² = 0.52), subsequent rm-ANOVAs were separately computed for shift and non-shift blocks. Within shift blocks, rm-ANOVA for reaction time indicated a significant endotoxin condition × word valence interaction (F_(1, 14)_ = 10.2, *p* = 0.006, ƞ_p_² = 0.42). This reflects that during endotoxemia, participants responded slower to sad (compared with happy target words) when they had to switch from positive to negative targets. In addition, endotoxin condition × mood interaction for mean reaction times within shift blocks approached significance (F_(1, 14)_ = 4.2, *p* = 0.06, ƞ_p_² = 0.23), indicating that participants tended to respond slower when sad mood was induced during the endotoxin condition, but to show faster responses when sad mood was induced in the placebo condition. Within non-shift blocks, analysis revealed significant effects for valence × mood condition interaction (F_(1, 14)_ = 13.8, *p* = 0.02, ƞ_p_² = 0.50; Table 1) as well as a for mood (F_(1, 14)_ = 6.4, *p* = 0.024, ƞ_p_² = 0.32, effect of mood condition), reflecting slower reaction times to happy words in neutral mood and to sad words in sad mood.

**Fig. 3:**
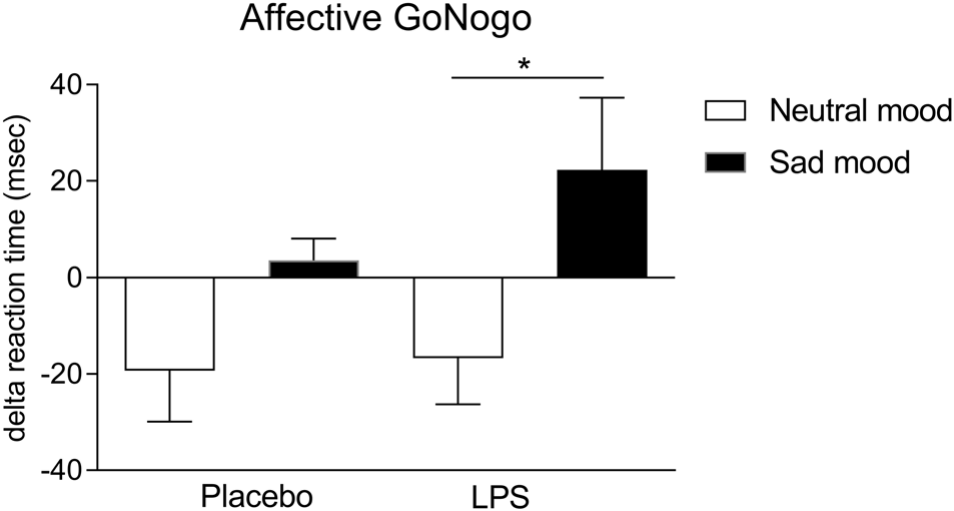
**Effects of endotoxin and negative mood on reaction time in the affective Go/Nogo task** Delta reaction times for positive vs. negative target words were computed according to Murphy et al.^27^ with positive scores indicating a tendency to respond slower and negative scores indicating a tendency to respond faster to sad targets. Differences in delta reaction time between the sad and neutral mood condition were significant only in the LPS condition (*p < 0.05, result of post-hoc paired *t*-test). For results of ANOVA, please see text

Finally, to further explore the effects of endotoxin and mood on attentional biases, we computed delta reaction times for positive vs. negative target words. Herein, rm-ANOVA displayed a significant effect of mood (F_(1, 14)_ = 7.5, *p* = 0.02, ƞ_p_² = 0.35), whereas the interaction effect was not significant (F_(1, 14)_ = 0.9, *p* = 0.37, ƞ_p_² = 0.06). Interestingly, post-hoc testing revealed that differences in delta reaction time between the sad and neutral mood condition were significant only in the LPS (t_(14)_ = −2.5, *p* = 0.027), but not in the placebo condition (Fig. 3). This finding supports that participants showed slower reaction times to sad vs. happy stimuli only if sad mood was induced during the LPS condition.

Neither for omission (F_(1, 14)_ = 1.1, *p* = 0.31, ƞ_p_² = 0.08) nor for commission (F_(1, 14)_ = 0.01, *p* = 0.94, ƞ_p_² = 0.01) errors (Table 1), an inflammation × mood interaction effect was observed. The number of omission errors was higher for negative compared with positive target words (F_(1, 14)_ = 4.8, *p* = 0.045, ƞ_p_² = 0.23), as well as in shift compared with non-shift blocks (F_(1, 14)_ = 38.5, *p* < 0.001, ƞ_p_² = 0.37, main effect; F_(1, 14)_ = 20.2, *p* = 0.001, ƞ_p_² = 0.59, valence × shift interaction).

To take possible inter-correlations between mood and sickness symptoms into account, analyses of GoNogo data were repeated with physical sickness symptom score as a covariate. Herein, we did not find significant effects in the full model (i.e., for the interaction of endotoxin × mood × valence × shift; data not shown). Within the shift condition, the endotoxin condition × word valence interaction remained as trend (F_(1, 14)_ = 4.6, *p* = 0.52, ƞ_p_² = 0.26), whereas the endotoxin condition × mood interaction was not significant (F_(1, 14)_ = 0.1, *p* = 0.90, ƞ_p_² = 0.01). Within non-shift blocks, the observed effects were no longer significant (F_(1, 14)_ = 2.6, *p* = 0.13, ƞ_p_² = 0.17, valence × mood condition interaction; F_(1, 14)_ = 0.7, *p* = 0.44, ƞ_p_² = 0.05, mood condition). Within the analysis of delta reaction times for positive vs. negative target words, the main effect of mood remained as a statistical trend (F_(1, 14)_ = 4.2, *p* = 0.06, ƞ_p_² = 0.24). Results of post-hoc testing, that is, differences in delta reaction time between the sad and neutral mood condition in the LPS, were still significant with the covariate (F_(13)_ = 5.4, *p* = 0.037, ƞ_p_² = 0.29). Finally, the results for omission and commission errors remained unchanged (data not shown).

## Discussion

Systemic inflammation is increasingly recognized as a risk factor for depression^1,2,4^. Disturbed affective cognition constitutes a core aspect in depression^19^, but has never been studied in the context of inflammation. This is the first study that combined experimental endotoxemia with a mood induction paradigm in order to assess effects on affective cognition. In this randomized, double-blind, placebo-controlled cross-over study conducted in healthy men, the application of low-dose endotoxin led to the expected transient increases in inflammatory markers, reflecting systemic immune activation as previously documented by our^9–11,29–31,37^ and other groups^13,14,16,17,38,39^. The mood induction paradigm successfully induced sad mood, with highest sadness ratings when sad mood was induced during endotoxemia. This is in line with an endotoxemia study revealing altered emotional responses in a social exclusion paradigm^18^. According to earlier findings that increased pro-inflammatory cytokines during endotoxemia correlated with symptoms of depression including dysthymia^9,10,40^, we observed that sadness ratings during endotoxemia were already increased at baseline, that is, *before* the induction of sad mood. Thus, the observed lower increase in sadness ratings in response to the mood induction paradigm during endotoxemia (as compared with the placebo condition) must be interpreted in light of higher baseline sadness ratings and may reflect ceiling effects.

Disturbed affective cognition constitutes a core aspect in depression^19^, but has thus far not been analyzed in the context of acute inflammation. To test affective cognition and to objectify cognitive biases, we applied the established AGNG task^27^. First, we observed a significant endotoxin condition by word valence interaction within switch blocks of the AGNG. During endotoxemia but not placebo treatment, participants responded slower to sad (compared with happy) target words when switching from positive to negative targets. In addition, participants tended to show slower reaction times when sad mood was induced during systemic inflammation. Given the absence of a main effect of endotoxin, this finding is clearly not a mere endotoxin effect on reaction time or cognitive functioning. Instead, it rather indicates a reduced ability to inhibit or reverse previously relevant associations during acute inflammation^27^. Second, sad mood induction impacted affective cognition, as supported by a significant mood condition by word valence interaction, along with a main effect of mood, on reaction time within non-shift blocks. This supports slower responses to sad words in the sad mood condition, irrespective of endotoxemia or placebo condition. Finally, we tested the interaction of endotoxin and sad mood conditions using an integrated delta outcome measure of affective cognition^27^. Results showed slower response times to sad versus happy stimuli only when sad mood was induced during endotoxemia. This novel finding supports that acute inflammation may augment the effect of sad mood on affective cognition, specifically on the processing of negative information. According to cognitive models of depression^21^, cognitive biases in information processing may ultimately contribute to depression risk^22^.

Of note, the finding that inflammation and sad mood led to *slower* responses to negative target words are at odds with our a priori hypothesis of a mood-congruent processing bias, that is, *faster* response times for negative compared with positive target words^27,41,42^. This hypothesis was built on evidence that depressed individuals^19^ demonstrated facilitated responses to sad or slowed responses to happy target words^27,41–43^. However, one study implementing the AGNG task in individuals with remitted depression also failed to show the expected negative response bias with respect to error rates^43^. Strikingly similar evidence of slower (rather than faster) responses to negative stimuli comes from studies using different attentional paradigms such as the emotional Stroop task. In this emotional analog of the Stroop color-naming test, depressed individuals consistently take longer to attend to negative stimuli^19,20^, a finding that was also reported after induction of sad mood in healthy volunteers (e.g., Isaac et al.^24^). Based on this body of evidence, it has been proposed that the prolonged and sustained processing of mood-congruent information reflects problems with disengagement from negative stimuli^23^, or interference effects with negative self-referent schemes triggered by negative emotions^44^. Hence, our findings may indicate a negative affective bias, characterized by a deeper processing of negative information.

The results of this study should be interpreted in the light of its strengths and limitations: experimental endotoxemia is an established model with high external validity, which has been instrumental in unraveling mechanisms involved in depression risk, including the complex and interdependent effects of immunological, physiological and behavioral changes induced by systemic immune activation. Combining this model with a mood induction paradigm allowed analyzing both main and interaction effects of inflammation and mood on affective cognition in a well-controlled experimental setting. On the other hand, the relatively small study sample bears the risk of limited statistical power and false negative results, although our choice of a within-group (cross-over) design reduces error variance and hence allows to detect even relatively small effects^45–47^. Although a cross-over design has important advantages as pointed out above, we cannot fully exclude that the results were influenced by order effects, especially in participants who received LPS during the first and placebo during the second visit. Order effects may result in unblinding of participants or in an unconscious or classical conditioning of LPS-induced immune responses or sickness symptoms. However, in separate (unpublished) analyses, we did not find evidence for order effects on immune-related or psychological data. Further, we included only male participants. Given the higher prevalence of depression in women on the one hand^48^, and a more pronounced responses to immune challenges^49^ including LPS administration^37^ in women on the other hand, future experiments should include women and specifically aim to analyze sex differences. Based on previous findings that women show greater LPS-induced changes in depressive symptoms^40^ and in neural responses to emotional stimuli^18^, one would expect more pronounced LPS effects on sadness rating and on affective Go/Nogo task reaction times in women. Endotoxin application induces physical sickness symptoms, which are demonstrably correlated with depressed mood^10^. To account for this, we herein conducted supplementary analyses of covariance to explore putative effects of sickness symptoms on the main outcome variables. We found clear evidence that sickness symptoms were associated with mood ratings in the mood induction paradigm. For affective cognition (i.e., Go/Nogo task), results were more heterogeneous, but results remained largely unchanged. Slight increases in *p*-values for Go/Nogo data, in some cases leading to loss of significance, should be interpreted in the light of low statistical power. Given that our main focus was to address effects of mood changes, we refrained from more complex analyses in this first proof-of-concept study. However, in follow-up studies in larger samples it will be important to disentangle possible interactions between mood, sickness symptoms and affective cognition.

Finally, we could implement only one test to evaluate affective cognition given time limitations due to dynamically changing cytokine profiles after endotoxin administration. Future studies should therefore consider different affective cognitive tasks such as the emotional Stroop task (e.g., Mitterschiffthaler et al.^44^) as well as non-affective (“cold”) cognitive tasks assessing executive functioning such as the Tower of London (e.g., Robinson et al.^33^) to complement and expand our findings.

## Conclusion

In summary, we demonstrated that inflammation and sad mood alter affective cognition in an affective Go/Nogo task. Acute inflammation impacts on the interplay between sad mood and affective cognition. These results may reflect a mood-congruency effect, with prolonged and sustained processing of mood-congruent information specifically when sad mood was induced during acute inflammation. Together, these factors may contribute to depression risk and call for future studies to disentangle specific neurobiological mechanisms both peripherally and within the brain.

## Electronic supplementary material

Supplementary Figure
