# Supplementary figures and images for: Effects of acute systemic inflammation on the interplay between sad mood and affective cognition
Source: Transl Psychiatry. 2017 Dec 11;7:1281. doi: 10.1038/s41398-017-0043-0 (PMC5802562; doi:10.1038/s41398-017-0043-0)

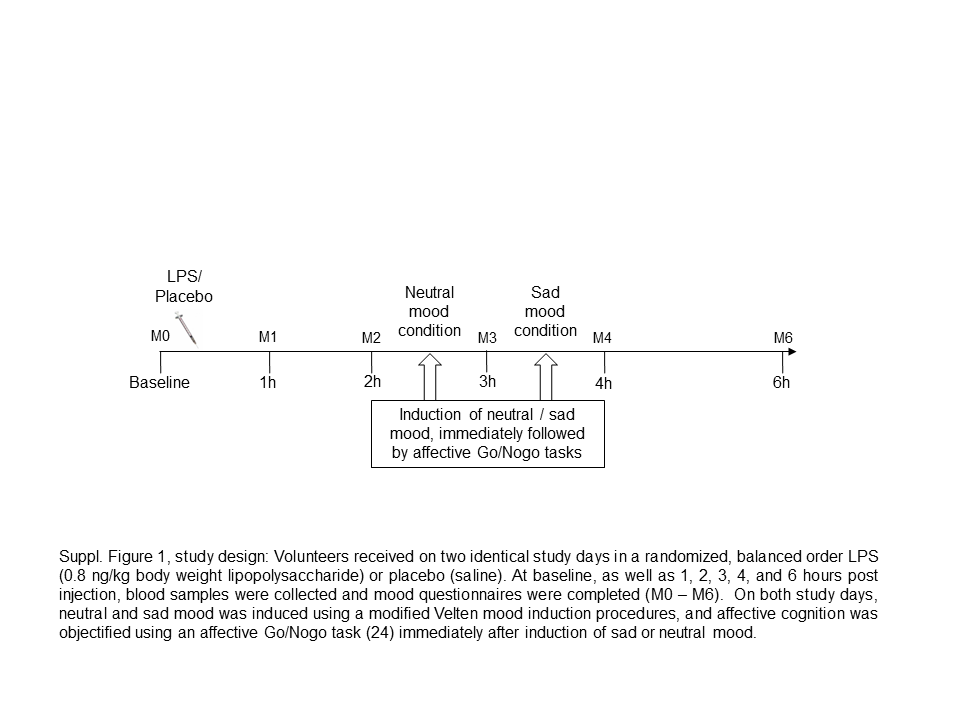

Supplement: Supplementary file 1 — Supplementary Figure [file 41398_2017_43_MOESM1_ESM.tif]
